# An effective introduction to structural crystallography using 1D Gaussian atoms

**DOI:** 10.1088/1361-6404/aa8188

**Published:** 2017-09-27

**Authors:** Emily Smith, Gwyndaf Evans, James Foadi

**Affiliations:** 1 Gonville and Caius College, Trinity Street, Cambridge, United Kingdom; 2 Diamond Light Source Ltd, Harwell Science and Innovation Campus, Didcot, United Kingdom; james.foadi@diamond.ac.uk

**Keywords:** crystallography, Fourier series, Fourier transform, anomalous scattering, computational methods for structural crystallography

## Abstract

The most important quantitative aspects of computational structural crystallography can be introduced in a satisfactory way using 1D truncated and periodic Gaussian functions to represent the atoms in a crystal lattice. This paper describes in detail and demonstrates 1D structural crystallography starting with the definition of such truncated Gaussians. The availability of the computer programme CRONE makes possible the repetition of the examples provided in the paper as well as the creation of new ones.

## 1. Introduction

X-ray crystallography is the most widely used technique to determine the shape of molecules at atomic resolution. The complexity of structures found has increased considerably since William Lawrence Bragg used diffraction images from various crystals to calculate the position of individual atoms in a crystal cell for sodium chloride, zinc sulphide and diamond [1]. Accordingly, the amount and difficulty of algorithms and calculations involved in data collection and structure solution have increased notably. This means that only researchers with strong quantitative and computational backgrounds are capable of designing and implementing adequate algorithms for processing data from the diffraction of x-ray crystals. Traditionally, physicists have been the largest group of investigators providing both innovation and calculating power in structural crystallography. With time, scholars from other sectors, chemistry and biology, have devoted substantial resources and manpower to the creation of vast computer program collections that can be easily accessed and used by scientists without the habitual quantitative background [2, 3]. Maintaining such systems and, especially, making sure they are subject to appropriate rounds of innovation requires the ability to recruit skilled personnel with adequate preparation in all quantitative aspects of structural crystallography. With its wide spectrum of subjects taught within condensed matter theory, physics is still the most represented among developers of methods in structural crystallography. This paper is hence generally aimed at physicists, with a particular eye to those engaged or interested in condensed matter theory. The paper is especially going to be valuable to students with an interest in becoming developers of methods and software in structural crystallography.

Structural crystallography is indeed a well known and mature subject. But it is riddled with specific jargon and requires familiarity with the concept of symmetry and especially all mathematical and computational aspects of the Fourier series and transform. Also, probability theory and statistical calculations are indispensable when processed data are used to determine the atomic positions. The learning curve is, therefore, quite steep. Things are furthermore complicated by the need to visualize and carry forward all calculations in 3D. The present paper aims at demonstrating that it is possible to learn all major and important quantitative concepts of structural crystallography using one, rather than three dimensions. The key to this demonstration is the representation of individual atoms with truncated and periodical 1D Gaussians, suitable to mimic crystal structures in the unit cell. The truncation of Gaussian functions is necessary in this contex to avoid infinite sums and integrals whose integrands are infinite sums in most calculations. A periodic and regular pattern with shifted Gaussians is, indeed, rigorously obtainable only if the number of Gaussians is infinite. Working with truncated Gaussians, in contrast, implies integrals with just one integrand, despite the crystal structure being represented by an infinite lattice.

1D crystallography is not, of course, a novel idea. It has recently been discussed and demonstrated in detail by Pinkerton [7] using carbon atoms, following an example similar to that of Stout and Jensen [8]. Such educational treatment of the subject is effective, but limited in scope. Challenges represented by most crystallographic calculations are barely addressed in these works. Quite differently, the original approach of the present paper lies in the extent to which all crystallographic ideas and formulas are developed, in the generality of 1D structures employed as examples (not simply made of carbon atoms), in the rigorous nature of the mathematics involved and, especially, in the creation of a specific computer program to carry out all calculations, plots and pictorial representations. An introduction to crystallography via 1D rather than 3D structures is valuable partly because it clarifies concepts and ideas, and because it makes mathematical formulas less cumbersome and diagrams more intuitive, thus allowing for a smoother initiation into a convoluted technical subject. Most importantly, integrals and Fourier series in 1D are well taught and received by students during the first two years of undergraduate studies. The greatest majority of students in quantitative subjects are certainly capable of understanding and interpreting the 1D mathematics of Fourier series and Fourier integrals. For this topic, the key passages in the calculations are all present in the 1D case. Moving up to 2D or 3D brings in an unnecessary pedagogical increase in complicated factorisations that only serve the purpose of making computations longer, heavier and, thus, less digestible. Another advantage of using a working model of a crystal structure and its Fourier counterparts in 1D, rather than 2D or 3D, is the speed and ease with which ideas and concepts in real space can be translated algorithmically and visually into reciprocal space. This fact is well known to methods developers in x-ray structural crystallography, when testing the initial correctness and effectiveness of their novel algorithms on 1D models (e.g. the infinite chain of carbon atoms).

In what follows, details and calculations are sketched in the main text and expanded in the appendices. A computer package, CRONE [4], developed for the R statistical platform [5] and freely distributed in the CRAN repository [6], can be downloaded for free and employed to closely follow the details of the exposure.

## 2. The crystal structure of 1D Gaussians

The most important mathematical feature of a crystal structure is its translational periodicity, for which the repeating portion of space is called *a unit cell*. Gaussians are desirable functions to represent the core electron density of atoms in the unit cell because they peak around the centre of their distribution, thus representing well the cloud of core electrons around the nucleus, and because they can be handled reasonably well from an analytical point of view. Unfortunately they are not periodic functions. One way to regain the lost periodicity is through the convolution of the Gaussian with the lattice function,

1 \begin{eqnarray*}{L}_{a}(x)\equiv \displaystyle \sum _{u=-\infty }^{+\infty }\delta (x-{ua}).\end{eqnarray*}

Indeed, a 1D crystal structure can be modelled as a function $\psi (x)$ equal to the convolution of the Gaussian centred at 0 and the lattice function:

2 \begin{eqnarray*}\psi (x) &amp; \equiv &amp; [{L}_{a}(x)]\,\ast \,\displaystyle \frac{1}{\sqrt{2\pi {\sigma }^{2}}}\exp \left(-\displaystyle \frac{{x}^{2}}{2{\sigma }^{2}}\right)\\ &amp; = &amp; {\displaystyle \int }_{-\infty }^{+\infty }\displaystyle \sum _{u=-\infty }^{+\infty }\delta (x^{\prime} -{ua})\displaystyle \frac{1}{\sqrt{2\pi {\sigma }^{2}}}\exp \left[-\displaystyle \frac{{(x^{\prime} -x)}^{2}}{2{\sigma }^{2}}\right]{\rm{d}}x^{\prime} \\ &amp; = &amp; \displaystyle \frac{1}{\sqrt{2\pi {\sigma }^{2}}}\displaystyle \sum _{u=-\infty }^{+\infty }\exp \left[-\displaystyle \frac{{(x-{ua})}^{2}}{2{\sigma }^{2}}\right].\end{eqnarray*}

It is easy to prove that the obtained result, the infinite summation of regularly shifted Gaussians, is a periodic function. Other properties can be worked out starting from its definition. But every calculation requires the summation of converging series, most of them involving the use of special functions (see appendix A). It is more convenient, pedagogically, to leave unaltered the analytic form of the Gaussian within one unit cell and to impose some kind of ‘forced’ periodicity whereby Gaussian tails are truncated so as to have their domain exactly equal to one period, i.e. equal to the unit cell length, *a*. In this section it will be shown how atoms and groups of atoms suitable for crystallographic calculations can be derived starting from the full non-periodic Gaussian function and ending with a truncated but periodic one. The resulting analytic formula is formally equivalent to a standard Gaussian function with a normalisation constant depending on cell's size and atom's width.

### 2.1. Gaussian atoms

A single 1D atom will be investigated first. As stated earlier, the following Gaussian function

**Figure 1.**
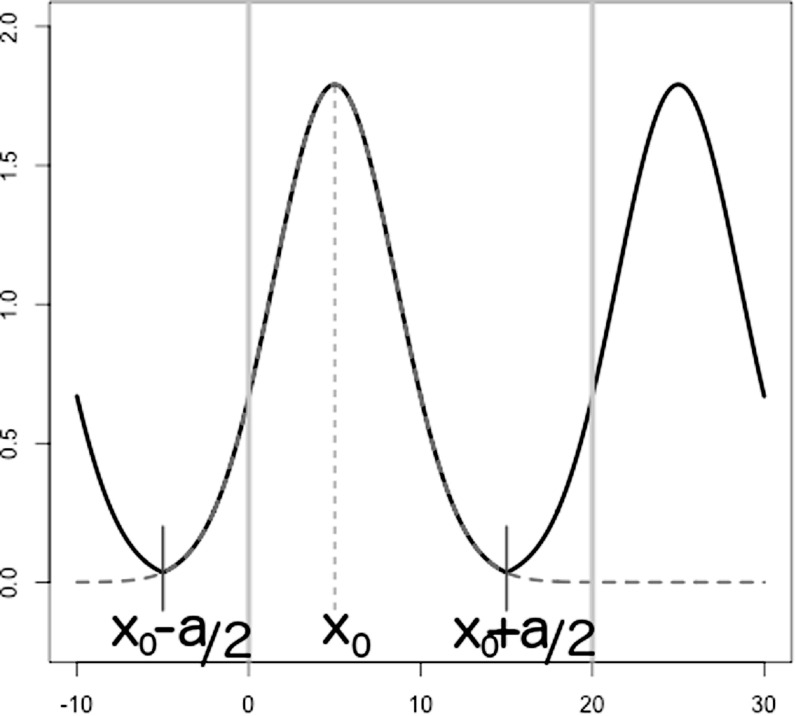
Procedure to turn a standard Gaussian into a periodic function. Initially the Gaussian is centred at *x*_0_ and its tails extend to $-\infty $ and $+\infty $ (red, dashed curve). This is then truncated at ${x}_{0}-a/2$ and ${x}_{0}+a/2$ (the two points marked with the two short blue segments). Copies of the truncated curve are created and shifted to the left and to the right by *na* for any integer *n* (black curve). In this specific example a = 20.

3 \begin{eqnarray*}\rho (x)\equiv \displaystyle \frac{1}{\sqrt{2\pi {\sigma }^{2}}}\exp \left[-\displaystyle \frac{{(x-{x}_{0})}^{2}}{2{\sigma }^{2}}\right]\end{eqnarray*}

extends from $-\infty $ to $+\infty $, is not periodical, but it is normalised, the area under its curve being 1. One way to transform it into a periodical function is to cut symmetrically its two tails and stick each end to the endpoints of two other identical Gaussians, truncated in the same way. In one dimension this is how to proceed (see figure 1). Both the left and right tails are truncated at a distance $a/2$ from the central peak, i.e. the two cuts occur at ${x}_{0}-a/2$ and ${x}_{0}+a/2$. Infinite copies of the truncated curve are, next, shifted to the left and to the right of *x*_0_ in discrete amounts equal to multiples of the unit cell length, *a*. The curve thus obtained is continuous but its first derivative is not, its discontinuities falling at ${x}_{0}\pm (2n+1)a/2,n=0,1,2,\,...$. The correct analytic form for this curve, limited to interval $[0,a]$, depends on whether *x*_0_ is in the first half or the second half of the unit cell:

(1) If $0\leqslant {x}_{0}\leqslant a/2$,
  4 \begin{eqnarray*}\left\{\begin{array}{ccc}\rho (x)=K\exp [-{(x-{x}_{0})}^{2}/(2{\sigma }^{2})] &amp; \mathrm{for} &amp; 0\leqslant x\leqslant {x}_{0}+a/2\\ \rho (x)=K\exp \{-{[x-({x}_{0}+a)]}^{2}/(2{\sigma }^{2})\} &amp; \mathrm{for} &amp; {x}_{0}+a/2\leqslant x\leqslant a\end{array}\right..\end{eqnarray*}
(2) If $a/2\leqslant {x}_{0}\leqslant a$,
  5 \begin{eqnarray*}\left\{\begin{array}{ccc}\rho (x)=K\exp \{-{[x-({x}_{0}-a)]}^{2}/(2{\sigma }^{2})\} &amp; \mathrm{for} &amp; 0\leqslant x\leqslant {x}_{0}-a/2\\ \rho (x)=K\exp [-{(x-{x}_{0})}^{2}/(2{\sigma }^{2})] &amp; \mathrm{for} &amp; {x}_{0}-a/2\leqslant x\leqslant a\end{array}\right..\end{eqnarray*}

*K* is the normalization constant calculated in appendix B and different from the usual $1/\sqrt{2\pi {\sigma }^{2}}$ because the Gaussian is truncated and the integration area of interest goes from 0 to *a*. It is, in fact, useful in 1D structural crystallography to require that the area under this curve be equal to *Z*, the atomic number. An auxiliary normalization function, *G*, depending on *a* and *σ*, can be conveniently introduced at this point. It is defined as

6 \begin{eqnarray*}G(a,\sigma )\equiv \displaystyle \frac{1}{\sqrt{2\pi {\sigma }^{2}}}{\left[\mathrm{erf}\left(\displaystyle \frac{a}{2\sqrt{2}\sigma }\right)\right]}^{-1},\end{eqnarray*}

where $\mathrm{erf}$ is the error function. The required normalization constant *K* is given by the following relation:

7 \begin{eqnarray*}K={ZG}(a,\sigma ).\end{eqnarray*}

The 1D Gaussian atom so defined depends on the two parameters *x*_0_ and *σ*, on the atomic number *Z* and on the unit cell length *a. x*_0_ is the atom's position, provided by the specific structure under investigation. *σ* is a measure of the size of the atomic number *Z* and the thermal vibration of the atom nucleus, more specifically the vibration of its centre. We will address both aspects shortly, but first it is important to understand that each atom needs to sit comfortably in the unit cell. This means that all atoms’ density should be contained inside the cell. Such condition is certainly met if the tail's truncation is carried at a certain integer quantity of standard deviations from the peak position. This integer *m*_*a*_ is given a default value of 5, but can, obviously, be increased or shortened. The following formula can be used to make sure that all the atoms are included:

8 \begin{eqnarray*}a={D}_{{\rm{M}}}+2{m}_{a}{\sigma }_{{\rm{M}}},\hspace{0.5cm}{D}_{{\rm{M}}}\equiv \mathop{\max }\limits_{{ij}}(| {x}_{i}-{x}_{j}| ),\hspace{0.1cm}{\sigma }_{{\rm{M}}}\equiv {\max }_{i}({\sigma }_{i}),\end{eqnarray*}

where *D*_M_ is the maximum distance between atoms in one unit cell; if only one atom is contained in the cell, ${D}_{{\rm{M}}}=0$. The default choice is, as said before, $a={D}_{{\rm{M}}}+10{\sigma }_{{\rm{M}}}$.

### 2.2. Atoms’ width and thermal vibration

It is reasonable for the atomic width to depend on the number of electrons *Z*. The width is also augmented if the atomic nucleus vibrates due to thermal motion because this corresponds to the stationary electron cloud being smeared over a larger region. From a quantitative point of view the ‘cold’ atom (no thermal vibrations) can be associated with a positive parameter ${\sigma }_{0}$. In the present article (and in the accompanying R package, CRONE) ${\sigma }_{0}$ is proportional to the square root of *Z*:

9 \begin{eqnarray*}{\sigma }_{0}={k}_{\sigma }\sqrt{Z},\end{eqnarray*}

with ${k}_{\sigma }=0.05$ by default. This ensures that all Gaussians in the unit cell have a shape that preserves individuality (each atom is separate from the others) because each atom's width scales up as the square root of the atomic number, rather than linearly with it. Atomic vibrations can be expressed as a probability of the nucleus being found around its position of equilibrium. An appropriate choice of probability is the normal distribution with variance traditionally indicated by the parameter *U*. The resulting electron density is well modelled as convolution between the ‘cold’ atom density and the probability distribution of the nucleus’ centre. In appendix C it is shown that the resulting width *σ* satisfies the following relation:

10 \begin{eqnarray*}{\sigma }^{2}={\sigma }_{0}^{2}+U,\end{eqnarray*}

and the normalized thermal Gaussian is given by the following expression:

11 \begin{eqnarray*}\rho (x)={ZG}(a,\sqrt{{\sigma }_{0}^{2}+U})\exp \left[-\displaystyle \frac{{(x-\mu )}^{2}}{2({\sigma }_{0}^{2}+U)}\right],\end{eqnarray*}

where *μ* can be either ${x}_{0}-a$, or *x*_0_ or ${x}_{0}+a$, as shown in equations (4) and (5), and *G* is the function defined by formula (6). The net effect of thermal vibration is clearly to broaden the Gaussian shapes, but the specific choice of normalization constant fixes the area under the curve equal to *Z*, as before.

### 2.3. Gaussian molecule and structure

A 1D crystal structure is obtained when one or more Gaussian atoms populate the unit cell. In the present paper we adopt a point of view similar to the one adopted in current structural crystallography, which approximates the electron density of the crystal structure with the sum of the electron density of all atoms included. If *ρ* is the crystal structure density and ${\rho }_{1},{\rho }_{2},\,\ldots ,\,{\rho }_{n}$ are the densities of the atoms composing the structure, then we will always assume that

12 \begin{eqnarray*}\rho (x)=\displaystyle \sum _{j=1}^{n}{\rho }_{j}(x-{x}_{j}),\hspace{0.5cm}0\leqslant x\leqslant a,\end{eqnarray*}

where *x*_*j*_ is the central peak position of atom *j*. This is not strictly true as interactions among valence electrons modify the region between nuclei. But relation (12) is approximately valid in the context of kinematic diffraction, where x-ray scattering is mostly affected by core electrons and where the time scales involved are much longer than the nuclei and electrons’ motion.

In general the unit cell can include one or more atoms. In addition, copies of the same atom can be the result of crystallographic symmetry operations. In three dimensions there exist 230 different types of symmetries. In 1D the scenario is greatly simplified, but the main features of symmetry are still retained as there are just two types called *P1* and $P\bar{1}$ ^3^ ^3^There exist certain patterns in 2D that exhibit a kind of linear symmetry described by the so-called *Frieze* groups. As the functions treated in our models are strictly 1D and have radial symmetry around atoms’ peaks, such symmetry patterns are not applicable in the present context. The description of symmetry in 1D can also be enriched when *quasi-crystals* are taken into account, but this topic is not essential for the introductory level of this paper.. The way symmetry is used in crystallography starts with the partition of unit cells in equal regions called *asymmetric units*. The atomic content in one asymmetric unit is equivalent to the that of any other asymmetric unit. Symmetry operations consider the coordinates of all atoms populating one asymmetric unit and produce equivalent atomic coordinates in all other asymmetric units. In 1D crystallography, with only two possible symmetries, the initial asymmetric unit can be chosen as either the whole unit cell, or as the segment between 0 and $a/2$. The two symmetries are quantitatively summarised in table 1. For example, suppose a P1̅ crystal structure with unit cell of length *a* = 10 has two atoms in an asymmetric unit at ${x}_{1}=2.5$ and ${x}_{2}=4.3$. Due to symmetry there will be two more atoms in the unit cell, one at ${x}_{3}=7.5$ and the other at ${x}_{4}=5.7$. *x*_3_ is the symmetry equivalent of *x*_1_ because −2.5 is outside the $[0,a]$ of the reference unit cell, but its translationally equivalent atom resides at $a-2.5=10-2.5=7.5$. Similarly, *x*_4_ is the symmetry equivalent of *x*_2_.

**Table 1.** Possible asymmetric units and symmetry operations for the two symmetry systems in 1D crystallography.

Symmetry type	Asymmetric units	Symmetry-related positions
P1	$0\leqslant x\leqslant a$	x
P1̅	$0\leqslant x\leqslant a/2$	x, −x
	$a/2\leqslant x\leqslant a$	

### 2.4. Atom's occupancy

In ideal crystal structures all atoms have translational equivalents in all unit cells. In general, though, crystal's imperfections mean that this is not always the case, and that some unit cells have missing atoms. This is taken care of theoretically with the idea of *occupancy*, a number between 0 and 1. An atom with occupancy equal to 1 will be found in the translationally equivalent positions across all unit cells. A smaller value of the occupancy means that the specific atom cannot be found in all unit cells. For instance, an atom with occupancy equal to 0.85 is found in roughly 85% of the crystal's unit cells.

The concept of occupancy is also useful when listing atoms located in the so-called *special position* of the cell, which is $a/2$ (in three dimensions there are more than one special position). An atom located at $x=a/2$ has a symmetry equivalent at exactly the same position; this is, obviously, the same atom. Thus, both atoms can be listed as a structure's content in the unit cell, as long as their occupancy is fixed at 0.5.

## 3. Linear molecules as models for 1D crystallography

1D crystallography can be demonstrated effectively using the structure of some existing linear molecules, as all their atoms lie approximately on a segment. Six molecules have been chosen to be used as examples in this paper. For each of them the interatomic distances have been searched on the Internet as published average bond lengths, but the coordinates have been placed arbitrarily in unit cells calculated using relation (8). Those cases in which one of the atoms acted as the inversion centre have been assigned space group P1̅. To characterise atomic thermal vibrations in these molecules we have used the so-called*B* factors, *B*, rather than the variances *U*, as customary in structural crystallography. They are defined as

13 \begin{eqnarray*}{B}=8{\pi }^{2}U.\end{eqnarray*}

The choice of *B* factors is for us arbitrary. We have, thus, decided to fix the variance for hydrogen at ${U}_{H}=80/(8{\pi }^{2}){\mathring{\rm A} }^{2}=10/{\pi }^{2}{\mathring{\rm A} }^{2}$, which corresponds to an approximate vibration of 1 Å around the equilibrium centre. For all other atoms it is reasonable to assume that such oscillations will have smaller amplitudes. This is heuristically justified by the energy equipartition theorem that distributes thermal energies in inverse proportion to atomic masses (atomic number, *Z*, in our case). All *B* factors assigned to the linear molecules can, thus, be calculated using the following equation:

14 \begin{eqnarray*}{U}_{j}=\displaystyle \frac{{U}_{H}}{{Z}_{j}}=\displaystyle \frac{10}{{\pi }^{2}{Z}_{j}},\hspace{0.5cm}{B}_{j}=8{\pi }^{2}{U}_{j}=\displaystyle \frac{80}{{Z}_{j}}.\end{eqnarray*}

So the* B* factors will be $80\hspace{0.25cm}{\mathring{\rm A} }^{2}$ for hydrogens, $80/6\approx 13.333\hspace{0.25cm}{\mathring{\rm A} }^{2}$ for carbons, $80/8=10\hspace{0.25cm}{\mathring{\rm A} }^{2}$ for oxygens, etc.

**Figure 2.**
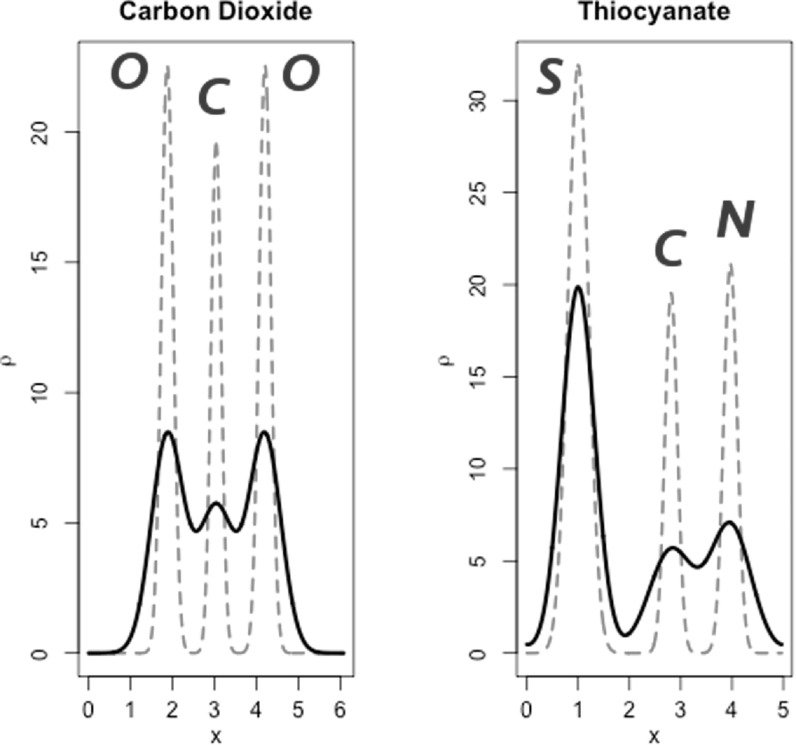
Density for two of the models reported in table 2, carbon dioxide and thiocyanate. The black curves correspond to all atoms having *B *factors as assigned in the table. The red dashed curve correspond to the same structures with ‘cold’ atoms, i.e. where all atoms have *B* factors equal to 0.

The six linear molecules are presented in table 2, with details of atomic positions and *B* factors. Plots of the density corresponding to carbon dioxide and thiocyanate in the table are shown in figure 2, superposed for comparison to the same structures with ‘cold’ atoms (all *B* factors equal to 0).

**Table 2.** 1D structures used as examples in this paper.

				Position in		
Molecule	Symmetry	Unit	Atomic	asymmetric		
Name		Cell (Å)	elements	unit (Å)	*B* factor (${\mathring{\rm A} }^{2}$)	Occupancy
Beryllium fluoride	P1̅	7.660	F	2.290	8.889	1.0
			Be	3.830	20.000	0.5

Carbon dioxide	P1̅	6.066	O	1.870	10.000	1.0
			C	3.033	13.333	0.5

Xenon difluoride	P1̅	11.674	F	3.837	8.889	1.0
			Xe	5.837	1.481	0.5

Nitronium	P1̅	6.014	O	1.857	10.000	1.0
			N	3.007	11.429	0.5

Cyanate	P1	3.833	O	0.707	10.000	1.0
			C	1.870	13.333	1.0
			N	3.126	11.429	1.0

Thiocyanate	P1	4.969	S	1.000	5.000	1.0
			C	2.813	13.333	1.0
			N	3.969	11.429	1.0

## 4. Fourier series and structure factors

The electron density for the full crystal, $\psi (x)$, is a periodical function. It can, therefore, be expressed as an infinite Fourier series:

15 \begin{eqnarray*}\psi (x)=\displaystyle \frac{1}{a}\displaystyle \sum _{h=-\infty }^{+\infty }{F}_{h}\exp \left(-2\pi {\rm{i}}h\displaystyle \frac{x}{a}\right),\end{eqnarray*}

where the complex expansion coefficient *F*_*h*_ in crystallography is called the *structure factor*. As explained by the theory of the Fourier series, this coefficient is calculated as an integral over one periodic unit or, in this case,

16 \begin{eqnarray*}{F}_{h}={\int }_{0}^{a}\psi (x)\exp \left(2\pi {\rm{i}}h\displaystyle \frac{x}{a}\right){\rm{d}}x\equiv {\int }_{0}^{a}\rho (x)\exp \left(2\pi {\rm{i}}h\displaystyle \frac{x}{a}\right){\rm{d}}x,\end{eqnarray*}

where the last passage is justified by the fact that in the interval $[0,a]$ function $\psi (x)$ coincides with $\rho (x)$, as defined by equations (4) and (5). The expression of the Fourier coefficient when there is only one atom in the unit cell is important and interesting because all other expressions can be built starting from it. We are, thus, looking to calculate integral (16) in which $\rho (x)$ is the piecewise function (4) or (5), with *K* given by expression (7). The full and detailed calculation is carried out in appendices D and E. It yields, for one atom:

17 \begin{eqnarray*}{F}_{h}=Z\exp \left(-2{\pi }^{2}{\sigma }^{2}\displaystyle \frac{{h}^{2}}{{a}^{2}}\right)\exp \left(2\pi {\rm{i}}h\displaystyle \frac{{x}_{0}}{a}\right).\end{eqnarray*}

The Fourier coefficient, or equivalent structure factor, is a complex quantity, as expected. Its amplitude, in this specific case of just one atom, is known as the *scattering factor* and indicated with the symbol *f*:

18 \begin{eqnarray*}f\equiv Z\exp \left(-2{\pi }^{2}{\sigma }^{2}\displaystyle \frac{{h}^{2}}{{a}^{2}}\right).\end{eqnarray*}

Its phase (or argument), contains *x*_0_ and is therefore connected to the atomic position. In the expression for the scattering factor *σ* is a generic quantity. For cold atoms $\sigma ={\sigma }_{0};$ for thermal atoms, as seen in formula (10), ${\sigma }^{2}={\sigma }_{0}^{2}+U$. We have, accordingly,

19 \begin{eqnarray*}{f}_{0}\equiv Z\exp \left(-2{\pi }^{2}{\sigma }_{0}^{2}\displaystyle \frac{{h}^{2}}{{a}^{2}}\right).\end{eqnarray*}

20 \begin{eqnarray*}f &amp; \equiv &amp; Z\exp \left(-2{\pi }^{2}{\sigma }^{2}\displaystyle \frac{{h}^{2}}{{a}^{2}}\right)\\ &amp; = &amp; Z\exp \left[-2{\pi }^{2}({\sigma }_{0}^{2}+U)\displaystyle \frac{{h}^{2}}{{a}^{2}}\right]\\ &amp; = &amp; Z\exp \left(-2{\pi }^{2}{\sigma }_{0}^{2}\displaystyle \frac{{h}^{2}}{{a}^{2}}\right)\exp \left(-2{\pi }^{2}U\displaystyle \frac{{h}^{2}}{{a}^{2}}\right).\end{eqnarray*}

This last expression can be re-written using *f*_0_, equation (19), and* B*, definition (13):

21 \begin{eqnarray*}f={f}_{0}\exp \left(-\displaystyle \frac{B}{4}\displaystyle \frac{{h}^{2}}{{a}^{2}}\right).\end{eqnarray*}

**Figure 3.**
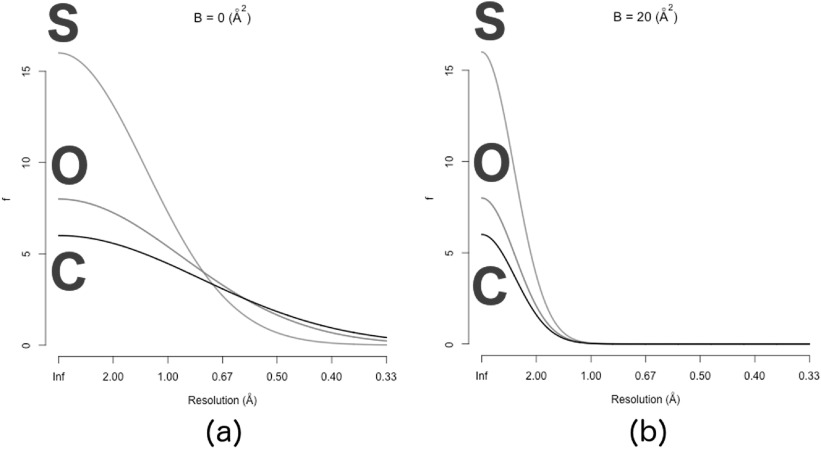
Scattering curves for sulphur (S), oxygen (O) and carbon (C). All curves are plotted against increasing resolution. (a) Curves obtained for cold atoms (*B* = 0); (b) curves obtained for thermal atoms with $B=20\hspace{0.25cm}{\mathring{\rm A} }^{2}$. It is very clear that the scattering factors of thermal atoms decay more rapidly than those of cold atoms.

The same result could have been obtained starting from the expression for the density of a thermal atom as a convolution because the Fourier integral of a convolution is the product of the individual Fourier integrals. The scattering factors of thermal atoms decay more rapidly than those of cold atoms because the exponential factor in equation (21) is a number smaller than 1 and decreases with increasing resolution (increasing values of the Miller index *h*). Examples of scattering curves plotted against increasing resolution, *h*/*a*, are shown in figure 3.

### 4.1. Structure factors of multiple atoms in the cell

When molecules, rather than one atom, are contained in the unit cell, the electron density is represented by equation (12). Integrals defining the structure factors are, in this case, equal to the sum of integrals for individual atoms. Therefore, the structure factor when more than one atom populates the unit cell is is also the sum of individual contributions:

22 \begin{eqnarray*}{F}_{h}=\displaystyle \sum _{j=1}^{n}{f}_{j}\exp \left(2\pi {\rm{i}}h\displaystyle \frac{{x}_{j}}{a}\right),\end{eqnarray*}

where considering equations (18), (19) and (21)

23 \begin{eqnarray*}{{f}}_{{j}}\equiv {{Z}}_{{j}}\exp \left(-2{{\pi }}^{2}{{\sigma }}_{{j}}^{2}\displaystyle \frac{{{h}}^{2}}{{{a}}^{2}}\right)\exp \left(-\displaystyle \frac{{{B}}_{{j}}}{4}\displaystyle \frac{{{h}}^{2}}{{{a}}^{2}}\right).\end{eqnarray*}

**Figure 4.**
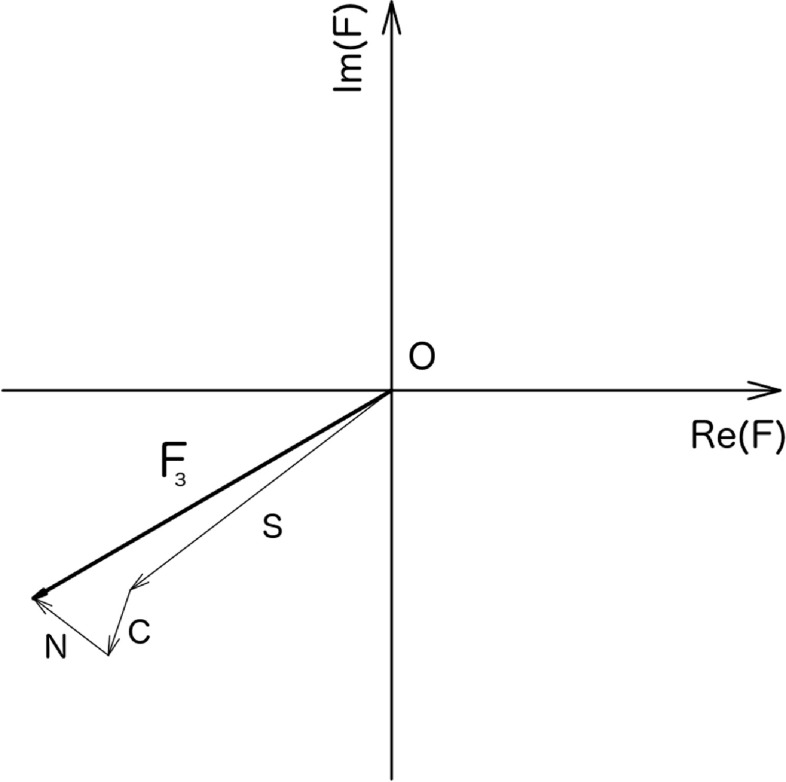
Structure factor *F*_3_ (Miller index *h* = 3) for thiocyanate, as sum of contributions by individual atoms in the complex Argand plane. The structure factor is mostly contributed by sulphur, as is clear from the figure.

From the above expressions it can clearly be seen that the structure factors of crystallographic structures composed of several atoms in the unit cell are the sum of individual complex numbers, each one of them having a length equal to the atom's scattering factor *f*_*j*_ and phase built out of the atom's position. A representation of this sum in the Argand plane for the thiocyanate structure in table 2 is shown in figure 4.

## 5. Anomalous scattering

The reason why most quantitative structural crystallography can be introduced using only one dimension is related to the fact that structure factors are defined in the complex plane irrespective of whether the underlying molecules are built in one, two or three dimensions. Once a method to calculate structure factors is available, a whole array of topics mimicking those in 3D crystallography can be explained and easily illustrated. In [7], for instance, a simple 1D structure is used to explain fundamental crystallographic concepts like unit-cell refinement, the Patterson function, E-values and direct methods, and structure refinement. These same concepts can, of course, be explained also with the general framework adopted to build 1D crystallographic structures using truncated Gaussian functions. In this paper these arguments are skipped and the remainder will instead be devoted to anomalous scattering, as this is of relevance to several techniques used in modern structural crystallography. What follows is a very cursory presentation of anomalous scattering; a very accessible but more in-depth introduction to the same subject can be found on the website maintained by Ethan Merritt at the Biomolecular Structure Centre, University of Washington [10].

Atoms scatter anomalously when the energy, and thus the wavelength, used is close to their resonant values. This means that in general crystals do not scatter anomalously or, better, that the magnitude of anomalous diffraction is negligible when compared to ordinary scattering. At specific wavelengths, though, certain atoms in the crystal resonate and the resulting diffracted beam includes significant anomalous contributions. Their particular nature is exploited for phasing, i.e. to find phases of the scattering factors. Ordinary diffraction is mathematically reflected in the usual factor appearing in equations (22) and (23). The expression for anomalous diffraction makes use of imaginary scattering factors in the following way:

24 \begin{eqnarray*}{F}_{h}=\displaystyle \sum _{j=1}^{n}({f}_{j}+{f}_{j}^{{\prime} }+{\rm{i}}{f}_{j}^{{\prime\prime} })\exp \left(2\pi {\rm{i}}h\displaystyle \frac{{x}_{j}}{a}\right).\end{eqnarray*}

**Figure 5.**
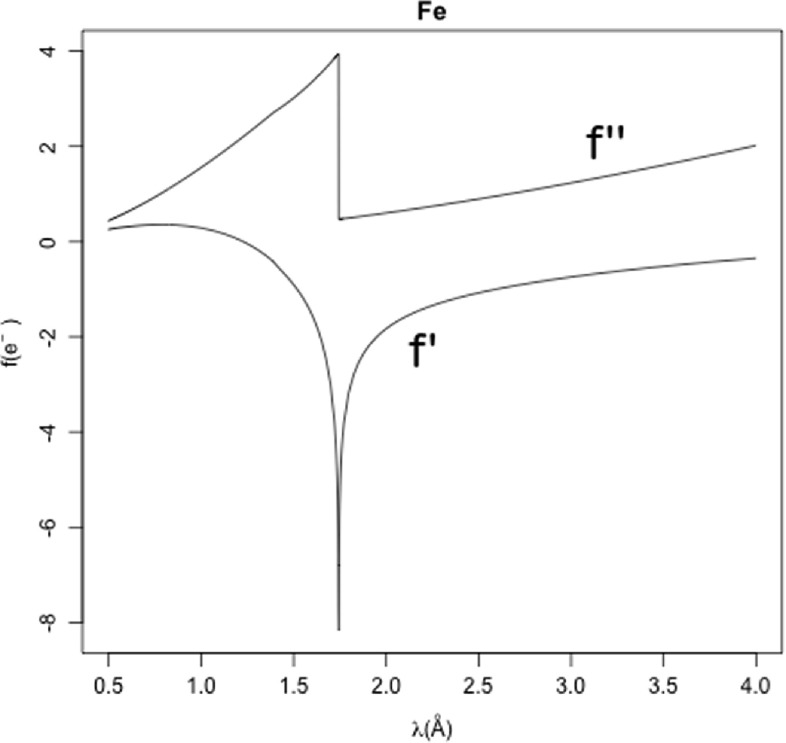
Curves of ${f}_{j}^{{\prime} }$ and ${f}_{j}^{{\prime\prime} }$ for iron atoms, as functions of wavelength. The resonant energy coincides with the dip shown in the ${f}_{j}^{{\prime} }$ curve.

The new scattering factor is equal to the ordinary, real, component plus a complex one, ${f}_{j}^{{\prime} }+{\rm{i}}{f}_{j}^{{\prime\prime} }$. Both the real and imaginary parts of this additional anomalous factor are virtually independent of resolution and only depend on wavelength. Values of ${f}_{j}^{{\prime} }$ and ${f}_{j}^{{\prime\prime} }$ for all atomic species and for several wavelengths have been calculated theoretically and can be easily tabulated. The values used in the CRONE package have been extracted from Ethan Merritt's site [10]. An example of ${f}_{j}^{{\prime} }$ and ${f}_{j}^{{\prime\prime} }$ as functions of wavelength for iron atoms is depicted at figure 5. The top curve describes ${f}_{j}^{{\prime\prime} }$, while the bottom curve describes ${f}_{j}^{{\prime} }$. In order to phase structures using anomalous scattering it is necessary to choose wavelengths close to the resonant energies (close to the dip of ${f}_{j}^{{\prime} }$) because in its neighbourhood the $f^{{\prime\prime} } $ anomalous contribution will be the largest, causing structure factors to change appreciably from their ordinary values.

The most important consequence that anomalous scattering has on structure factors is the breaking of the so-called *Friedel*’*s law*, according to which amplitudes of structure factors with opposite Miller indices are identical, and phases are opposite of each other. For structures not scattering anomalously in any appreciable way, such amplitudes will not be identical, due to experimental errors, but very similar. When some atoms in the structure scatter anomalously, the same related amplitudes are expected to be systematically different. It is on such difference that methods for structure factors’ phase estimate are based, as illustrated in the rest of this section.

Given a Miller index, *h*, and its opposite, $-h$, Friedel's law states that the structure factor associated with *h* is the complex conjugate of the structure factor associated with $-h$:

25 \begin{eqnarray*}{F}_{-h}={F}_{h}^{* }\hspace{0.5cm}\iff \hspace{0.5cm}| {F}_{-h}| =| {F}_{h}| \hspace{0.2cm}\mathrm{and}\hspace{0.2cm}{\varphi }_{-h}=-{\varphi }_{h}.\end{eqnarray*}

When contributions from the anomalous scattering become important, though, Friedel's Law is not valid any longer and in general the following will be true:

26 \begin{eqnarray*}| {F}_{-h}| \ne | {F}_{h}| .\end{eqnarray*}

In this case the pair corresponding to opposite Miller indices, called a Bijvoet pair, is separated into a *plus* and a *minus* part, indicated as ${F}^{+}$ and ${F}^{-}$. One of the methods used to phase crystal structures with anomalous phasing employs Bijvoet pairs to create a special density map called an *anomalous Fourier difference* map (or anomalous difference in short). This density map has amplitudes equal to the absolute square difference of each Bijvoet pair, and all phases equal 0. If the anomalous difference map is indicated as ${\rho }_{{\rm{ano}}}(x)$, then

27 \begin{eqnarray*}{\rho }_{{\rm{ano}}}(x)=\displaystyle \frac{1}{a}\displaystyle \sum _{h=-\infty }^{+\infty }{{\rm{\Delta }}}_{{\rm{ano}}}^{2}(h)\exp \left(-2\pi {\rm{i}}h\displaystyle \frac{x}{a}\right),\end{eqnarray*}

where

28 \begin{eqnarray*}{{\rm{\Delta }}}_{{\rm{ano}}}(h)\equiv | | {F}^{+}| -| {F}^{-}| | .\end{eqnarray*}

It can be shown that the anomalous difference map is equivalent to a corrupted Patterson map in which some of the prominent peaks correspond to distances between the anomalous scatterers, e.g. peaks between heavy atoms [11]. Once these distances have been measured, an initial, approximate model of the structure, made of anomalous scatterers, can be suggested. An updated Fourier transform calculated with the experimental structure factors’ amplitudes and phases from the initial model, should show improved positions for the anomalous scatterers, but also peaks corresponding to atoms not yet determined. The updated density map will be closer to the true density map if the selected peaks from the anomalous Fourier difference are the correct ones. The updated density can, in turn, be used to confirm or reject the positions of the anomalous scatterers and to select new peaks for the atoms missing from the initial approximate model of the structure. Old and new peaks will form a new structural model used to carry out another Fourier cycle (observed amplitudes and calculated phases). This sort of Fourier recycling provides atomic positions converging to the correct structure, if the initial anomalous scatterers have been properly selected. A specific example will make the procedure clearer.

**Figure 6.**
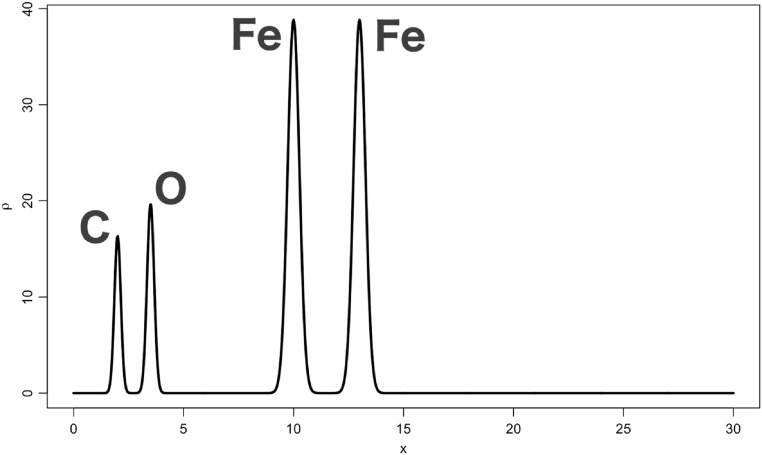
Atoms corresponding to the structure used for the anomalous phasing example in section 5.

**Figure 7.**
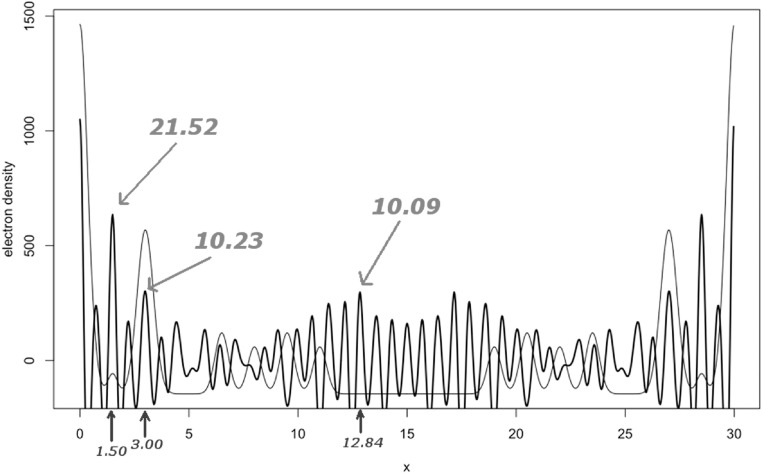
Patterson map (thin curve) and anomalous difference map (thick curve) for the example structure in section 5. The three highest peaks of the anomalous difference map are pointed at with arrows; their position in the cell is annotated at the bottom of the figure. These three peaks correspond to three anomalous scatterer models with which phasing is attempted.

**Figure 8.**
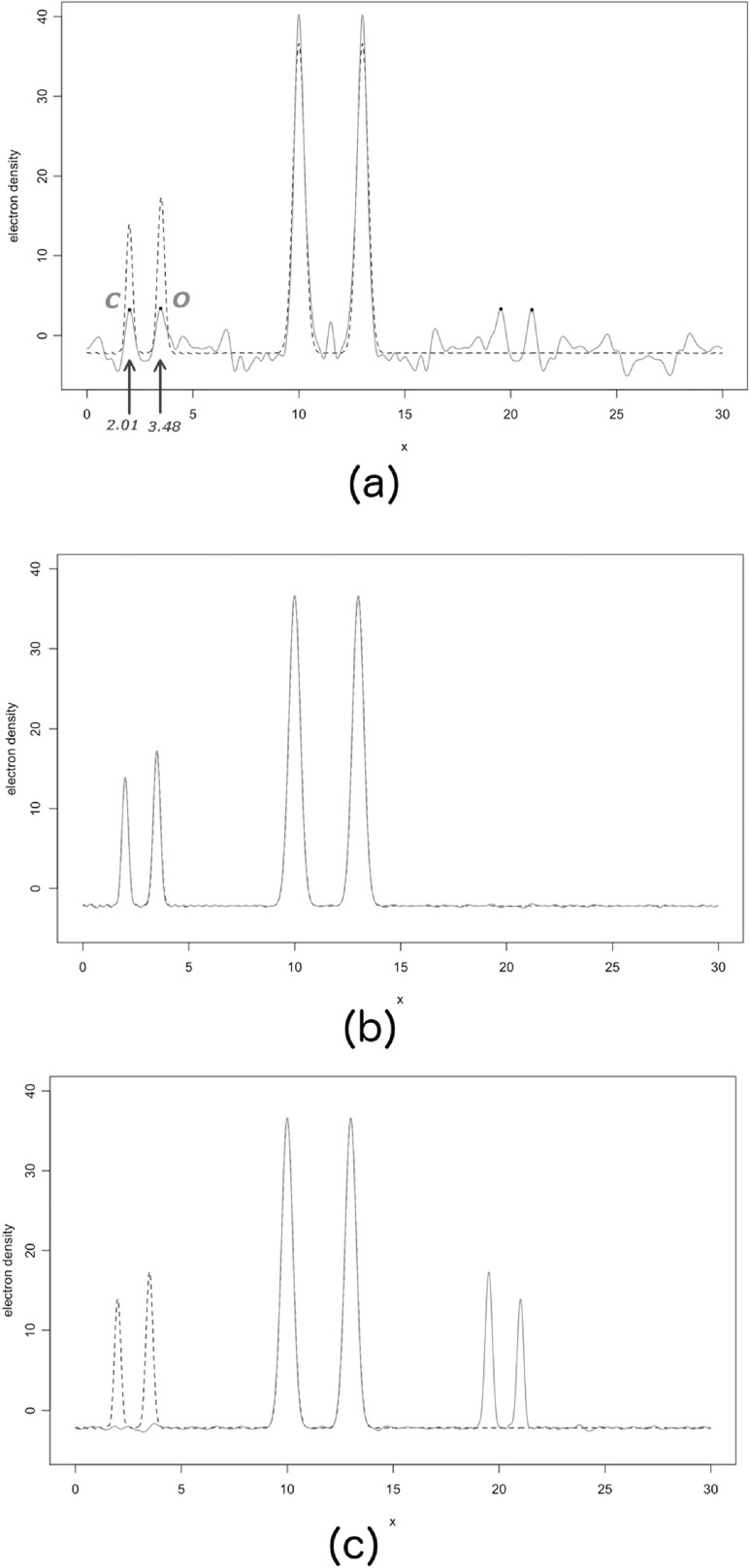
Approximate electron densities (red, full curves) compared with the final correct density (black, broken curve), during the process of phase determination. The first approximate density is shown in part (a) of the figure. The correct position of the two iron atoms is reflected in the height of the two largest peaks. Four more peaks are possible candidates for the carbon and oxygen in the final structure. The correct ones are indicated with C and O and their inclusion in the updated structural model yields the density in part (b) of the figure. Selection of the other two peaks, instead, leads to the incorrect structure, as shown in part (c).

**Figure 9.**
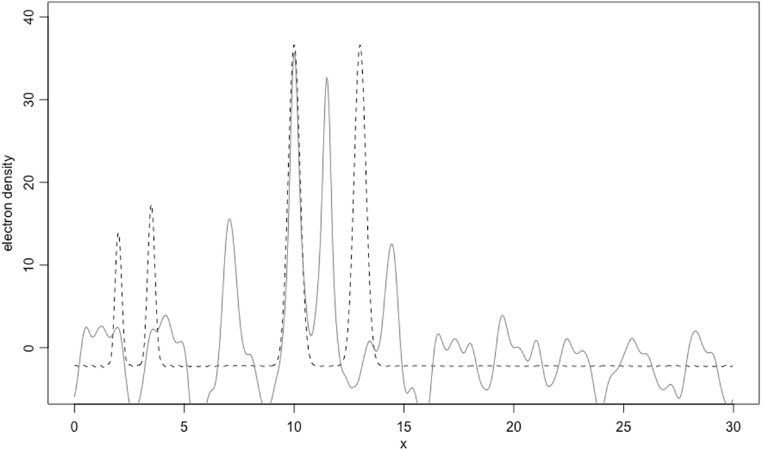
Approximate electron densities (red, full curve) compared with the final correct density (broken curve), when the interatomic distance from the anomalous difference map is not the correct one.

Consider a P1 structure made of one carbon, one oxygen and two iron atoms at positions:

\begin{eqnarray*}{x}_{1}=2.0,\hspace{0.1cm}{x}_{2}=3.5,\hspace{0.1cm}{x}_{3}=10.0,\hspace{0.1cm}{x}_{4}=13.0\end{eqnarray*}

in a unit cell of side *a* = 30 Å. All atoms are kept at a very low temperature, corresponding to a *B* factor equal to 0.5 Å^2^. All atoms in this structure are represented in figure 6. Structure factors have been computed for positive and negative Miller indices up to *h* = 80. Their amplitudes for the first five Bijvoet pairs and corresponding averages, taken to be equal to the observed amplitudes, are tabulated in table 3. The anomalous difference map, calculated with the anomalous differences just obtained, is displayed as thick curve in figure 7, jointly with the Patterson map (thin curve). Some of the highest peaks in the anomalous difference map (with the exclusion of the origin peak, which is always the highest peak) correspond to the interatomic distance between anomalous scatterers. Of the three highest peaks only the one with a height of 10.23 and located at $x=3.00$ coincides with a prominent Patterson peak: it is, therefore, the most likely candidate to indicate the distance between the two iron atoms. We can place two iron atoms anywhere in the unit cell (because of the arbitrariness of the cell's origin), as long as their distance is 3.00 Å, i.e. the position of the chosen peak in the anomalous difference map. The two iron atoms arranged in this way form an initial model for the complete structure. To make the comparison with the known structure easier, the origin of the unit cell has been located so to have the two iron atoms placed at 10 Å and 13 Å. Phases calculated from this model are used jointly with the observed amplitudes to generate the first approximate electron density map. This is shown in figure 8(a), overlapped to the final correct structure. The approximate density has, obviously, high peaks at the iron position because the chosen interatomic distance from the anomalous Fourier was the correct one. More peaks are present in the density, though. Among the four highest ones there should be peaks corresponding to the carbon and oxygen atoms missing from the initial model. If we select the two peaks at position $x=2.01$ (carbon atom) and $x=3.48$ (oxygen atom), the updated electron density appears as in figure 8(b), basically matching the density for the correct structure. If the other two peaks (at positions $x=19.53$ and $x=21.00$) are used, instead, the updated density does not resemble the correct one (figure 8(c)). If the peak selected in the anomalous difference map had been the highest one, at position $x=1.50$, the initial approximate electron density would have been the same as the one depicted at figure 9. This electron density does not resemble the one for the correct structure, with the exception of the peak in correspondence with the only correctly placed atom at *x* = 10.

**Table 3.** Miller indices, Bijvoet pairs amplitudes, observed amplitudes and anomalous differences for the first five structure factors computed in relation to the structure used in the anomalous phasing example (section 5).

${\bf{h}}$	${{\bf{F}}}^{+}$	${{\bf{F}}}^{-}$	${{\bf{F}}}_{{\rm{obs}}}$	${{\boldsymbol{\Delta }}}_{{\rm{ano}}}({\bf{h}})$
1	37.65	41.65	39.65	4.00
2	24.74	22.11	23.43	2.62
3	35.14	33.14	34.14	2.00
4	20.65	22.30	21.48	1.65
5	9.90	9.93	9.92	0.03

## 6. The CRONE package

A package for the R statistical platform [5], named CRONE (CRystallography in ONE dimension) [4], has been developed to carry out all calculations explained in this paper, and other operations useful for structural crystallography in 1D. CRONE is freely downloadable directly from any CRAN repository [6] or from the GitHub site [4]. This package is comprised of functions, datasets and tables sufficient to create and execute numerous examples of crystallographic calculations using 1D Gaussian atoms. A few common crystallography operations, explained and demonstrated with the help of CRONE, are included in appendix F. A variety of additional examples can be found in the package’s help pages as well as in an R-specific descriptive document available within the package and known as *vignettes*.

## 7. Conclusions

Amongst the most interesting and attractive aspects of structural crystallography, the mathematics of Fourier series and Fourier transforms play a dominant role in the community of physicists. Many of the most important features in this area can be explained and illustrated analytically making use of Gaussian functions. These concepts are very familiar to physics undergraduates and graduates, as they are taught in several modules. The 1D introduction to structural crystallography described in the present paper provides, therefore, an excellent starting point to captivate students’ attention and to steer their interest in this important topic across both the physical and biological sciences. Such an introduction makes use of truncated and periodical 1D Gaussians to represent atoms in a crystal lattice, both visually and quantitatively. Furthermore, the close parallel between the complex Fourier coefficients for both 1D and 3D structures facilitates the transition to modern crystallographic jargon. Students that learn 1D crystallography as explained in this paper will have an immediate grasp of both the qualitative and quantitative aspects of the discipline. The paper also introduces and demonstrates a new package in the R environment for statistics, CRONE, that makes any calculation within 1D crystallography possible and easy. Its use is a valid aid in classroom teaching and enables the generation of innumerable examples and exercises at various levels.

## Figures and Tables

**Figure F1.**
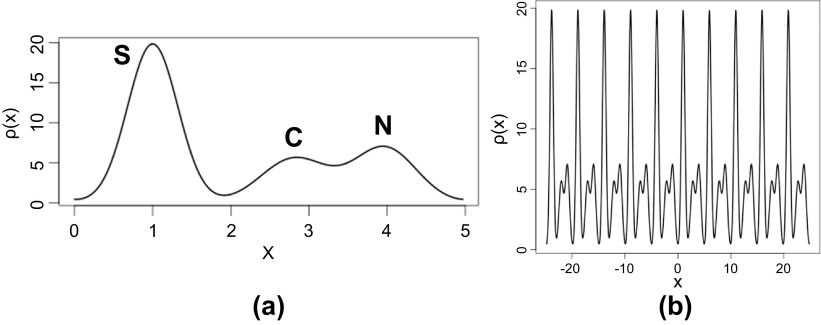
1D density inside (a) one and (b) multiple unit cells, for thiocyanate. The three peaks correspond to the central position of sulphur, carbon and nitrogen.

**Figure F2.**
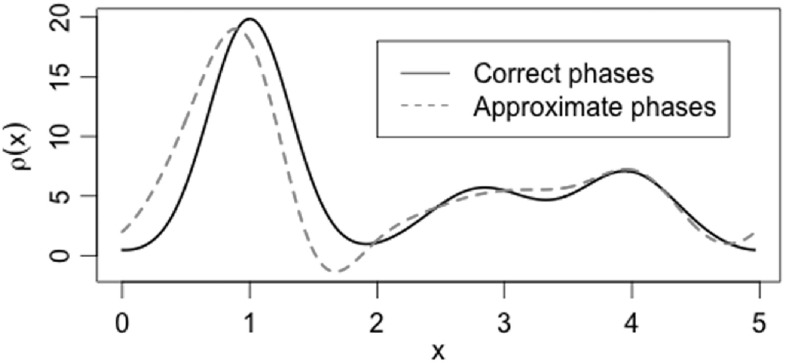
Correct (full line) and approximate (dash line) density for thiocyanate. The approximate density is the result of a Fourier synthesis having correct structure factor amplitudes, but only an approximate estimate of the correct structure factors phases.

**Figure F3.**
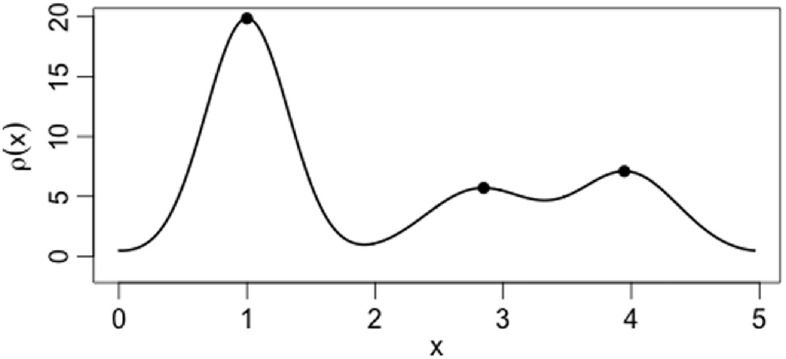
Peaks found in the correct density, revealing the position of the three atoms of thiocyanate. The values found, $x=0.999,x=2.847$ and $x=3.945$ are very close to the published values in table 2.

**Figure F4.**
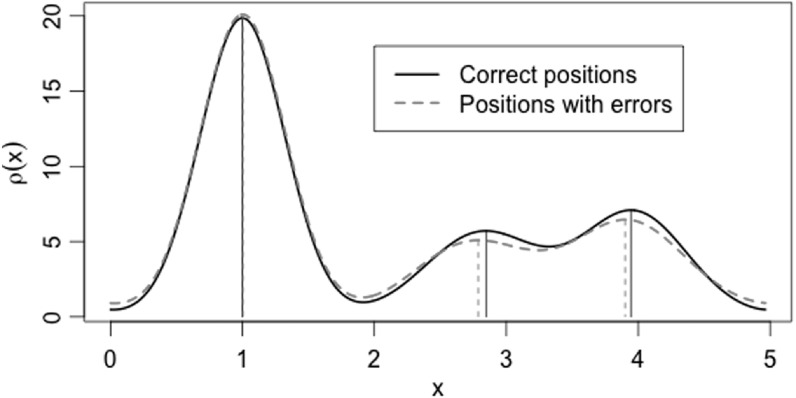
Discrepancy between correct density for thiocyanate and density calculated with modified structure factors’ amplitudes affected by errors in the coordinates.

## Appendix A. Infinite crystal structures with full Gaussians: some results

Let us consider the simple case of a 1D structure made out of the regular repetition of one atom with atomic number represented by the integer *Z*, and where the spacing between atoms is *a*, equal to the cell length. Without loosing generality we can place atomic centres at the lattice points, $...\,-2a,-a,0,+a,+2a,\,...$. The structure is represented by the expression

A.1 \begin{eqnarray*}\psi (x)=\displaystyle \frac{Z}{\sqrt{2\pi {\sigma }^{2}}}\displaystyle \sum _{u=-\infty }^{+\infty }\exp \left[-\displaystyle \frac{{(x-{ua})}^{2}}{2{\sigma }^{2}}\right]\end{eqnarray*}

The following points help define the character of this function.

(i) $\psi (x)$ is periodical, with period a.
   We can see this by computing $\psi (x+{ka})$ and proving that this is equal to $\psi (x)$, where *k* is any integer:
  \begin{eqnarray*}\psi (x+{ka}) &amp; = &amp; \displaystyle \frac{Z}{\sqrt{2\pi {\sigma }^{2}}}\displaystyle \sum _{u=-\infty }^{+\infty }\exp \left[-\displaystyle \frac{{(x+{ka}-{ua})}^{2}}{2{\sigma }^{2}}\right]\\  &amp; = &amp; \displaystyle \frac{Z}{\sqrt{2\pi {\sigma }^{2}}}\displaystyle \sum _{u=-\infty }^{+\infty }\exp \left\{-\displaystyle \frac{{[x-(u-k)a]}^{2}}{2{\sigma }^{2}}\right\}.\end{eqnarray*}
  The quantity *u* − *k* is an integer running from $-\infty $ to $+\infty ;$ therefore, calling ${u}^{{\prime} }\equiv u-k$ we have:
  A.2 \begin{eqnarray*}\psi (x+{ka})=\displaystyle \frac{Z}{\sqrt{2\pi {\sigma }^{2}}}\displaystyle \sum _{{u}^{{\prime} }=-\infty }^{+\infty }\exp \left[-\displaystyle \frac{{(x-{u}^{{\prime} }a)}^{2}}{2{\sigma }^{2}}\right]\equiv \psi (x).\end{eqnarray*}
(ii) The value of $\psi ({ka})$ is analytically given by an auxiliary Jacobi special function.
  The value of $\psi (x)$ at any lattice point *ka*, where *k* is an integer, can be approximated using the Jacobi auxiliary theta function ${\theta }_{3}$, which is a complex function defined in the context of elliptic integrals. In detail:
  A.3 \begin{eqnarray*}\psi ({ka})=\psi (0+{ka})=\psi (0)\end{eqnarray*}
  because the function is periodic. Therefore,
  A.4 \begin{eqnarray*}\psi ({ka})=\displaystyle \frac{Z}{\sqrt{2\pi {\sigma }^{2}}}\displaystyle \sum _{u=-\infty }^{+\infty }\exp \left(-\displaystyle \frac{{a}^{2}}{2{\sigma }^{2}}{u}^{2}\right).\end{eqnarray*}
  The following property applies to infinite sums of Gaussian-like terms:
  A.5 \begin{eqnarray*}\displaystyle \sum _{u=-\infty }^{+\infty }\exp (-{{Ku}}^{2})={\theta }_{3}(0,\exp (-K)),\end{eqnarray*}
  where ${\theta }_{3}(z,q)$ is the Jacobi third auxiliary function [9] with *nome* equal to *q*. Using property (A.5) in equation (A.4) brings us to the following result:
  A.6 \begin{eqnarray*}\psi ({ka})=\displaystyle \frac{Z}{\sqrt{2\pi {\sigma }^{2}}}{\theta }_{3}\left(0,\exp \left(-\displaystyle \frac{{a}^{2}}{2{\sigma }^{2}}\right)\right).\end{eqnarray*}
  It might be possible to compute $\psi (x)$ for values of *x* different from *ka*, but such calculation necessarily involves a deeper and thorough use of Jacobi theta functions, a topic beyond the scope and aim of this paper.
(iii) ${\psi }({x})$ is normalised in the interval corresponding to the unit cell. The area under the curve represented by $\psi (x)$ is, obviously, infinite because such an area is the infinite summation of the finite and positive area in any interval corresponding to the unit cell. It is, though, important to verify that the area corresponding to one unit cell is finite and to calculate its value. The area to be computed is:
  A.7 \begin{eqnarray*}{\rm{a}}{\rm{rea}} &amp; = &amp; {\displaystyle \int }_{-a/2}^{+a/2}\psi (x){\rm{d}}x\\ &amp; = &amp; \displaystyle \frac{1}{\sqrt{2\pi {\sigma }^{2}}}\displaystyle \sum _{u=-\infty }^{+\infty }{\displaystyle \int }_{-a/2}^{+a/2}\exp \left[-\displaystyle \frac{{(x-{ua})}^{2}}{2{\sigma }^{2}}\right]{\rm{d}}x.\end{eqnarray*}
  Using the substitution $(x-{ua})/(\sqrt{2}\sigma )\equiv t$, expression (A.7) becomes
  A.8 \begin{eqnarray*}{\rm{a}}\mathrm{rea} &amp; = &amp; \displaystyle \frac{1}{\sqrt{\pi }}\displaystyle \sum _{u=-\infty }^{+\infty }{\displaystyle \int }_{(-u-1/2)[a/(\sqrt{2}\sigma )]}^{(-u+1/2)[a/(\sqrt{2}\sigma )]}\exp (-{t}^{2}){\rm{d}}t\\ &amp; = &amp; \displaystyle \frac{1}{2}\displaystyle \sum _{u=-\infty }^{+\infty }\left\{\mathrm{erf}\left[\left(-u+\displaystyle \frac{1}{2}\right)\displaystyle \frac{a}{\sqrt{2}\sigma }\right]-\mathrm{erf}\left[\left(-u-\displaystyle \frac{1}{2}\right)\displaystyle \frac{a}{\sqrt{2}\sigma }\right]\right\},\end{eqnarray*}
  where $\mathrm{erf}$ is the *error function* defined as:
  A.9 \begin{eqnarray*}\mathrm{erf}(x)=\displaystyle \frac{2}{\sqrt{\pi }}{\int }_{0}^{x}\exp (-{t}^{2}){\rm{d}}t.\end{eqnarray*}
  The infinite summation can be re-written as the limit of a finite summation:
  A.10 \begin{eqnarray*}{\rm{a}}{\rm{r}}{\rm{e}}{\rm{a}}=\displaystyle \frac{1}{2}\mathop{\mathrm{lim}}\limits_{n\to +\infty }\displaystyle \sum _{u=-n}^{+n}\left\{\mathrm{erf}\left[\left(-u+\displaystyle \frac{1}{2}\right)\displaystyle \frac{a}{\sqrt{2}\sigma }\right]-\mathrm{erf}\left[\left(-u-\displaystyle \frac{1}{2}\right)\displaystyle \frac{a}{\sqrt{2}\sigma }\right]\right\}.\end{eqnarray*}
  After long, tedious, but not difficult simplifications, the expression for the area becomes:
  A.11 \begin{eqnarray*}{\rm{a}}\mathrm{rea}=\displaystyle \frac{1}{2}\mathop{\mathrm{lim}}\limits_{n\to +\infty }\left\{\mathrm{erf}\left[\left(-n+\displaystyle \frac{1}{2}\right)\displaystyle \frac{a}{\sqrt{2}\sigma }\right]-\mathrm{erf}\left[\left(-n-\displaystyle \frac{1}{2}\right)\displaystyle \frac{a}{\sqrt{2}\sigma }\right]\right\},\end{eqnarray*}
  and using the parity of the error function,
  A.12 \begin{eqnarray*}{\rm{a}}\mathrm{rea}=\displaystyle \frac{1}{2}\mathop{\mathrm{lim}}\limits_{n\to +\infty }2\ \mathrm{erf}\left[\left(n+\displaystyle \frac{1}{2}\right)\displaystyle \frac{a}{\sqrt{2}\sigma }\right]=1.\end{eqnarray*}
  Thus, the area of the infinite sum of Gaussians within a unit cell is finite and equal to 1. From a qualitative point of view this is sensible because, although there are infinite contributions that raise the curve upwards in a unit cell, such contributions are infinitesimally smaller and smaller, so that the final infinite sum converges.

## Appendix B. Normalization constant for the truncated Gaussian

Given the following function,

B.1 \begin{eqnarray*}\rho (x)=K\exp \left[-\displaystyle \frac{{(x-{x}_{0})}^{2}}{2{\sigma }^{2}}\right]\end{eqnarray*}

we want to compute that value of the constant *K* that makes the area under $\rho (x)$, between 0 and *a*, equal to *Z*, the atomic number. Calculations are equivalent if the integral is computed between $-a/2$ and $+a/2$ for the Gaussian centred at the origin. This yields the following expression:

B.2 \begin{eqnarray*}K{\int }_{-a/2}^{+a/2}\exp [-{x}^{2}/(2{\sigma }^{2})]{\rm{d}}x=Z\end{eqnarray*}

or, equivalently,

B.3 \begin{eqnarray*}2K{\int }_{0}^{+a/2}\exp [-{x}^{2}/(2{\sigma }^{2})]{\rm{d}}x=Z.\end{eqnarray*}

If *x* is replaced with the new integration variable $t\equiv x/\sqrt{2{\sigma }^{2}}$, the expression becomes

B.4 \begin{eqnarray*}2\sqrt{2{\sigma }^{2}}K{\int }_{0}^{a/(2\sqrt{2}\sigma )}\exp (-{t}^{2}){\rm{d}}t=Z.\end{eqnarray*}

Now, the error function, $\mathrm{erf}(x)$ is defined as

B.5 \begin{eqnarray*}\mathrm{erf}(x)\equiv \displaystyle \frac{2}{\sqrt{\pi }}{\int }_{0}^{x}\exp (-{t}^{2}){\rm{d}}t.\end{eqnarray*}

Thus, equation (B.4) can be re-written as

B.6 \begin{eqnarray*}\sqrt{2\pi {\sigma }^{2}}K\mathrm{erf}\left(\displaystyle \frac{a}{2\sqrt{2}\sigma }\right)=Z,\end{eqnarray*}

which yields

B.7 \begin{eqnarray*}K=Z\displaystyle \frac{1}{\sqrt{2\pi {\sigma }^{2}}}{\left[\mathrm{erf}\left(\displaystyle \frac{a}{2\sqrt{2}\sigma }\right)\right]}^{-1}.\end{eqnarray*}

Considering definition (6) for function *G*, *K* is therefore given by equation (7).

## Appendix C. Truncated Gaussian for a thermal atom

The general form of a Gaussian for the ‘cold’ atom in equations (4) and (5) is

C.1 \begin{eqnarray*}{\rho }_{0}(x)={ZG}(a,{\sigma }_{0})\exp [-{(x-\mu )}^{2}/(2{\sigma }_{0}^{2})],\hspace{0.5cm}0\leqslant x\leqslant a,\end{eqnarray*}

where *μ* is either ${x}_{0}-a$, or *x*_0_, or ${x}_{0}+a$. When the atomic nucleus vibrates due to an increase of energy (thermal vibration) the whole electron cloud is dragged along in this motion and the net macroscopic effect is a smearing of the cloud over a larger region. Mathematically the effect can be quantified through a convolution between density (C.1) and a normalized Gaussian probability with mean 0 and variance *U*:

C.2 \begin{eqnarray*}p(x)=\displaystyle \frac{1}{\sqrt{2\pi U}}\exp [-{x}^{2}/(2U)],\hspace{0.5cm}-\infty \lt x\lt +\infty .\end{eqnarray*}

The Gaussian density of the thermal atom is, therefore, defined as

C.3 \begin{eqnarray*}\rho (x)={\int }_{-\infty }^{+\infty }p(x-x^{\prime} ){\rho }_{0}(x^{\prime} ){\rm{d}}x^{\prime} ,\hspace{0.5cm}0\leqslant x\leqslant a.\end{eqnarray*}

The calculation is easier to illustrate if only one period of the atom, in the interval $[0,a]$, is used for ${\rho }_{0}$, while the probability is defined in the whole $(-\infty ,+\infty )$ interval. The full set of periodical atoms can be used to derive the final result, albeit with some pedagogical difficulty. If expressions (C.1) and (C.2) are inserted into expression (C.3) for the convolution, the result is:

C.4 \begin{eqnarray*}\rho (x)=\displaystyle \frac{{ZG}(a,{\sigma }_{0})}{\sqrt{2\pi U}}{\int }_{-\infty }^{+\infty }\exp \left\{-\left[\displaystyle \frac{{(x^{\prime} -x)}^{2}}{2U}+\displaystyle \frac{{(x^{\prime} -\mu )}^{2}}{2{\sigma }_{0}^{2}}\right]\right\}{\rm{d}}x^{\prime} .\end{eqnarray*}

Most of the algebraic manipulation needed now concerns the argument in square brackets. This should be transformed into the sum of two new quantities, one of which, including ${(x-\mu )}^{2}$, can be taken out of the integral. In fact, after a long, tedious but not difficult, series of algebraic passages, the argument inside the square brackets becomes

C.5 \begin{eqnarray*}\left(x^{\prime} -\displaystyle \frac{x{\sigma }_{0}+\mu U}{{\sigma }_{0}^{2}+U}\right)/\left(2\displaystyle \frac{U{\sigma }_{0}^{2}}{{\sigma }_{0}^{2}+U}\right)+{(x-\mu )}^{2}/[2({\sigma }_{0}^{2}+U)].\end{eqnarray*}

The first term contains $x^{\prime} $, the integration variable. After integration, this term gives rise to a number that multiplies all factors outside the integral in (C.4). The second term is untouched so that expression (C.4) becomes

C.6 \begin{eqnarray*}\rho (x)=K^{\prime} \exp \left[-\displaystyle \frac{{(x-\mu )}^{2}}{2({\sigma }_{0}^{2}+U)}\right].\end{eqnarray*}

$K^{\prime} $ will not, in general, yield a properly normalized function. We have the freedom, though, to adopt a different constant in the definition of *ρ*. We can, in fact, use for *ρ* the same normalization used in equation (7) because this guarantees the area under the curve to be *Z*. The functional part of *ρ* is identical to that of ${\rho }_{0}$ if ${\sigma }_{0}$ is replaced by $\sqrt{U+{\sigma }_{0}^{2}}$. Thus, the final, properly normalized, Gaussian density for thermal atoms is

C.7 \begin{eqnarray*}\rho (x)={ZG}(a,\sqrt{{\sigma }_{0}^{2}+U})\exp \left[-\displaystyle \frac{{(x-\mu )}^{2}}{2({\sigma }_{0}^{2}+U)}\right],\hspace{0.5cm}0\leqslant x\leqslant a.\end{eqnarray*}

For *U* = 0 this function correctly returns ${\rho }_{0}$.

## Appendix D. Fourier coefficient of a truncated Gaussian

We would like to calculate

D.1 \begin{eqnarray*}{F}_{h}={\int }_{0}^{a}\rho (x)\exp \left(2\pi {\rm{i}}h\displaystyle \frac{x}{a}\right){\rm{d}}x,\end{eqnarray*}

where $\rho (x)$ is given by the piecewise functions (4) or (5), and where *K* in such expressions is defined in equation (7). Even though there are four different analytic expressions in the definition of *ρ*, there is no need to carry out four separate integrations. Each branch of the piecewise density include terms of the following form:

D.2 \begin{eqnarray*}K\exp \left[-\displaystyle \frac{{(x-\mu )}^{2}}{2{\sigma }^{2}}\right].\end{eqnarray*}

Furthermore, the important feature of the integration interval used to calculate the Fourier coefficients is its width, *a*. This means that such interval does not have to be centred necessarily on $x=a/2$. It is, in fact, convenient to centre it on $x=\mu $. The integral needed to compute the Fourier coefficients, thus, has the form

D.3 \begin{eqnarray*}{F}_{h}=K{\int }_{\mu -a/2}^{\mu +a/2}\exp \left[-\displaystyle \frac{{(x-\mu )}^{2}}{2{\sigma }^{2}}\right]\exp \left(2\pi {\rm{i}}h\displaystyle \frac{x}{a}\right){\rm{d}}x,\end{eqnarray*}

with *μ* any of the values *x*_0_, ${x}_{0}+a$ or ${x}_{0}-a$. To solve integral (D.3) the new variable $y\equiv x-\mu $ is used. The substitution yields

D.4 \begin{eqnarray*}{F}_{h}=K\exp \left(2\pi {\rm{i}}h\displaystyle \frac{\mu }{a}\right){\int }_{-a/2}^{+a/2}\exp \left(-\displaystyle \frac{{y}^{2}}{2{\sigma }^{2}}\right)\exp \left(2\pi {\rm{i}}h\displaystyle \frac{y}{a}\right){\rm{d}}y.\end{eqnarray*}

To avoid using integration techniques in the complex plane and evaluating the error function for complex numbers it is, at this point, convenient to separate (D.4) into real and imaginary parts. The integral of the imaginary part involves integration within a symmetric interval of an odd function (product of a sine and a Gaussian); this integral is, therefore, zero. We are, thus, left with the following:

D.5 \begin{eqnarray*}{F}_{h}=K\exp \left(2\pi {\rm{i}}h\displaystyle \frac{\mu }{a}\right){\int }_{-a/2}^{+a/2}\cos \left(2\pi h\displaystyle \frac{y}{a}\right)\exp \left(-\displaystyle \frac{{y}^{2}}{2{\sigma }^{2}}\right){\rm{d}}y.\end{eqnarray*}

The integral in equation (D.5) is computed in appendix E and is equal to

D.6 \begin{eqnarray*}\sqrt{2\pi {\sigma }^{2}}\mathrm{erf}\left(\displaystyle \frac{a}{2\sqrt{2{\sigma }^{2}}}\right)\exp \left(-2{\pi }^{2}{\sigma }^{2}\displaystyle \frac{{h}^{2}}{{a}^{2}}\right)\equiv \displaystyle \frac{1}{G(a,\sigma )}\exp \left(-2{\pi }^{2}{\sigma }^{2}\displaystyle \frac{{h}^{2}}{{a}^{2}}\right).\end{eqnarray*}

So, given that $K={ZG}(a,\sigma )$, the Fourier coefficient becomes

D.7 \begin{eqnarray*}{F}_{h} &amp; = &amp; {ZG}(a,\sigma )\exp \left(2\pi {\rm{i}}h\displaystyle \frac{\mu }{a}\right)\displaystyle \frac{1}{G(a,\sigma )}\exp \left(-2{\pi }^{2}{\sigma }^{2}\displaystyle \frac{{h}^{2}}{{a}^{2}}\right)\\ &amp; = &amp; Z\exp \left(-2{\pi }^{2}{\sigma }^{2}\displaystyle \frac{{h}^{2}}{{a}^{2}}\right)\exp \left(2\pi {\rm{i}}h\displaystyle \frac{\mu }{a}\right).\end{eqnarray*}

When *μ* is replaced with either of *x*_0_, ${x}_{0}+a$ or ${x}_{0}-a$ equation (D.7) coincides with equation (17).

## Appendix E. Integrals of trigonometric and Gaussian functions

The purpose of the present appendix is to calculate an integral of the form

E.1 \begin{eqnarray*}I(h)\equiv {\int }_{-a/2}^{+a/2}\cos \left(2\pi h\displaystyle \frac{y}{a}\right)\exp (-{{by}}^{2}){\rm{d}}y.\end{eqnarray*}

This is the integral contained in expression (D.5), where

E.2 \begin{eqnarray*}b=\displaystyle \frac{1}{2{\sigma }^{2}}.\end{eqnarray*}

In order to calculate a derivative inside the integral, let us assume *h* to be a continuous, real variable. Equation (E.2) can be seen as a function of *h*, and its derivative calculated with respect to *h*,

E.3 \begin{eqnarray*}\displaystyle \frac{{\rm{d}}I(h)}{{\rm{d}}h}=-{\int }_{-a/2}^{+a/2}\displaystyle \frac{2\pi }{a}y\sin \left(2\pi h\displaystyle \frac{y}{a}\right)\exp (-{{by}}^{2}){\rm{d}}y.\end{eqnarray*}

After an integration by parts, the expression becomes

E.4 \begin{eqnarray*}\displaystyle \frac{{\rm{d}}I(h)}{{\rm{d}}h}=-\left\{{\left[-\displaystyle \frac{\pi }{{ab}}\exp (-{{by}}^{2})\sin \left(2\pi h\displaystyle \frac{y}{a}\right)\right]}_{-a/2}^{+a/2}\right.\end{eqnarray*}

E.5 \begin{eqnarray*}\left.+{\displaystyle \int }_{-a/2}^{+a/2}\displaystyle \frac{\pi }{{ab}}2\pi \displaystyle \frac{h}{a}\cos \left(2\pi h\displaystyle \frac{y}{a}\right)\exp (-{{by}}^{2}){\rm{d}}y\right\}.\end{eqnarray*}

The integral in this expression is $I(h)$ times a constant. The calculation leads to the following first-order differential equation:

E.6 \begin{eqnarray*}\displaystyle \frac{{\rm{d}}I(h)}{{\rm{d}}h}+\left(\displaystyle \frac{2{\pi }^{2}}{{a}^{2}b}h\right)I(h)=\displaystyle \frac{2\pi }{{ab}}\exp \left(-\displaystyle \frac{{a}^{2}b}{4}\right)\sin (\pi h).\end{eqnarray*}

For values of *h* in an infinitesimal neighbourhood of any integer, the right-hand side of the equation is very close to zero and assumes the following form:

E.7 \begin{eqnarray*}\displaystyle \frac{{\rm{d}}I(h)}{{\rm{d}}h}+\left(\displaystyle \frac{2{\pi }^{2}}{{a}^{2}b}h\right)I(h)=0,\end{eqnarray*}

whose solution is

E.8 \begin{eqnarray*}I(h)=C\exp \left(-\displaystyle \frac{{\pi }^{2}}{b}\displaystyle \frac{{h}^{2}}{{a}^{2}}\right).\end{eqnarray*}

The integration constant *C* can be found when *h* = 0 is used in (E.8), using also definition (E.1):

E.9 \begin{eqnarray*}C=I(0)={\int }_{-a/2}^{+a/2}\exp (-{{by}}^{2}){\rm{d}}y=\sqrt{\displaystyle \frac{\pi }{b}}\mathrm{erf}\left(\displaystyle \frac{a\sqrt{b}}{2}\right).\end{eqnarray*}

Therefore, the integral (E.1) is

E.10 \begin{eqnarray*}I(h)=\sqrt{\displaystyle \frac{\pi }{b}}\mathrm{erf}\left(\displaystyle \frac{a\sqrt{b}}{2}\right)\exp \left(-\displaystyle \frac{{\pi }^{2}}{b}\displaystyle \frac{{h}^{2}}{{a}^{2}}\right).\end{eqnarray*}

If we use the value in (E.2) in place of *b* we obtain the result for the integral in expression (D.5), yielding the final result (D.6).

## Appendix F. Demonstration of the CRONE package

Throughout this section knowledge of R is assumed. The reader wishing to learn more about this popular computational platform can find a substantial amount of introductory material on the R official website [5]. More tutorials can also be found in the website of one of the authors [12].

### F.1. Calculation and display of a 1D structure

The six 1D structures available in CRONE, or any other existing or arbitrary linear molecules, can be calculated and displayed directly in direct space, or by making the transition via reciprocal space. The starting points for both calculations are:

• Length of unit cell, a (in angstroms);
• Space group symbol, SG (‘P1’ or ‘P-1’);
• Vector x0 of atomic positions in the unit cell;
• Vector Z of atomic numbers corresponding to all atoms in the cell;
• Vector B of *B* factors for all atoms in the cell;
• Vector occ of atomic occupancies

#### F.1.1. Data preparation

These quantities can be created *ad hoc* in a completely arbitrary way, or loaded in the working directory from one of the six structures included in the package. Whichever way data are created, they can be arranged in the standard format used throughout the package, a named list, known as sdata, with its six elements equal to those displayed above. Here we will assume to be working with thiocyanate (see table 2). When data from this structure are loaded in the R working space, the following sdata object is created:

**Table:**

> sdata $\lt -$ load_structure(‘thiocyanate’)
> print(sdata)
$a
[1] 4.969
$SG
[1] ‘P1’
$x0
[1] 1.000 2.813 3.969
$Z
[1] 16 6 7
$B
[1] 5.000 13.333 11.429
$occ
[1] 1 1 1

This 1D structure has three atoms in a unit cell of length $a=4.969\,\mathring{\rm A} $. They are sulphur (S, Z = 16) at position $x=1.000$, carbon (C, Z = 6) at position $x=2.813$ and nitrogen (N, Z = 7) at position $x=3.969$. Their *B* factors are, respectively, 5.000, 13.333 and 11.429. All have occupancies 1. The symmetry for this structure is P1, which means there is no symmetry.

#### F.1.2. Calculation and display in direct space

The CRONE function to calculate density corresponding to 1D structures is structure_gauss. The calculation is carried out at every point of a regular grid. The result is a list, rho, with two vectors, the regular grid x and the density values rr. Both vectors are jointly used to display the structure (see figure F1(a)):

**Table:**

> rho $\lt -$ structure_gauss(sdata, N = 1000)
> plot (rho$x, rho$rr, type=‘l’, xlab = ‘x’, ylab = expression(rho))

Function structure_gauss can also be used to display the density in multiple unit cells, simply modifying the starting grid x (see figure F1(b)):

**Table:**

> x $\lt -$ seq(−5∗sdata$a,5∗sdata$a, length=1000)
> rho $\lt -$ structure_gauss(sdata, x=x)
> plot (rho$x,rho$rr, type=‘l’, xlab=‘x’, ylab=expression(rho))

#### F.1.3. Calculation and display in reciprocal space

The density displayed at figure F1(a) can also be calculated as Fourier series if the structure factors (i.e. the Fourier coefficients) are computed first. The function in charge of structure factors calculation in CRONE is called strufac. We will need to decide in advance how many Fourier components are required. The remaining part of the input is equivalent to the one used in the calculation for direct space, that is the information on the structure, sdata. The final result of the calculation is a named list with two elements, called Fmod and Fpha, which are vectors corresponding to the structure factors’ amplitudes and phases, respectively. Their location in both vectors corresponds to the location in vector hidx of input indices (Miller indices). Due to the way 1D truncated Gaussians are normalised, the fundamental component (hidx=0) of the Fourier series is always going to be equal to the sum of atomic numbers of all atoms in the unit cell (29 for this specific structure):

**Table:**

> hidx $\lt -$ 0 : 20
> ftmp $\lt -$ strufac(hidx, sdata)
> print(names(ftmp))
[1] ‘Fmod’ ‘Fpha’
> print(ftmp$Fmod [1 : 4])
[1] 29.000000 6.289007 12.500798 9.582935
> print (ftmp$Fpha [1 : 4])
[1] 0.00000 75.66567 147.71590−149.88296
> sum (sdata$Z)
[1] 29

The structure factors can be used to calculate a Fourier summation for all points of a grid x representing the unit cell. The function of CRONE available to carry out this so-called *Fourier synthesis* is fousynth:

**Table:**

> N $\lt -$ 1000 # Number of grid points in the unit cell
> rtmp $\lt -$ fousynth (sdata$a, ftmp$Fmod, ftmp$Fpha, hidx, N)
> x $\lt -$ rtmp$x
> rho $\lt -$ rtmp$rr

The vectors x and rho obtained are the same as those giving the density in figure F1(a).

### F.2. Approximate phases and peak search

The main goal of structural crystallography is to find the positional coordinates of all atoms in a given structure. The quantity that can be calculated using experimental data is the electron density. Atomic coordinates correspond to the peaks in the electron density. Positional precision and accuracy depend, of course, on the calculated electron density quality, this, in turn, being dependent on the precision and accuracy of all estimated structure factors phases. Amplitudes and phases from the structure factors of thiocyanate (see section F.1.3) can be computed using strufac; we call the two vectors created as Fm and Fph, respectively. As phases are not obtained experimentally, but are estimated with some degree of uncertainty, we can simulate approximate ones, Fapp, starting from their correct value, Fph and adding a small random error:

**Table:**

$\gt \#$ Amplitudes and phases of correct coefficients
> Fm $\lt -$ ftmp$Fmod
> Fph $\lt -$ ftmp$Fpha
$\gg \#$ Simulation of approximate phases
> Fapp[1] $\lt -$ Fph[1] # The h=0 component is untouched
> set.seed(9195) # Fix simulated random numbers
> Fapp[2 : 21] $\lt -$ Fph[2 : 21] + rnorm(19, mean=0, sd=0.4)
>
># Approximate density > rhoapp $\lt -$ fousynth (sdata$a, Fm, Fapp, hidx, N)

The Fourier synthesis obtained using experimental amplitudes Fm and approximated phases Fapp will be in general slightly different from the correct density (see figure F2).

Next, we need to find peak coordinates in the density obtained. In CRONE this is achieved using the function local_maxima. The peaks found in the correct density are shown in figure F3 and have values 0.999, 2.847 and 3.945, very close to the values in table 2. The second peak is not found in the approximate density. Maxima are displayed only for the first and third atom. They occur at 0.889 and 3.955. Starting with these values and cycling between structure factors and density, a convergence to correct values is eventually reached.

### F.3. Errors in structure factors

Amplitudes from calculated structure factors are never exactly met in experiments. Experimental quantities are different from their theoretical values for many reasons. In CRONE function sfobs simulates errors of two types for the structure factor amplitudes,

• Poissonian counting errors
• Positional atomic coordinate errors

*Poissonian counting errors* are due to the fluctuations in the number of photons scattered along a given direction (thus corresponding to certain Miller indices). The net effect of these fluctuations is to alter the structure factors theoretical amplitude, producing slightly smaller or larger values, depending on the amount of photons. Fluctuations in the value of sfobs are simulated as random generations of poissonian variates with mean equal to the theoretical structure factor's amplitude. The final value is calculated as average of all simulated amplitudes and the error as the corresponding standard deviation. The number of simulated deviates is controlled by the parameter ntrialP (default 100). As an example let us compare theoretical and simulated amplitudes for Miller indices hidx=1,…,5, for thiocyanate: the *positional atomic coordinate errors* simply simulates errors of the atomic centres around their correct values as Gaussian errors with mean zero and standard deviation controlled by the parameter vx0err. The underlying structure is only approximately equal to the correct structure and, accordingly, the corresponding structure factors’ amplitudes are slightly different from their calculated values. The example shown in the following simulate errors in the atomic coordinates for thiocyanate; the standard deviation used for the simulation is vx0err=0.4 Å. Poisson counting errors have been cancelled by using ntrialP=0: the structure corresponding to structure factors calculated with errors just in the atomic coordinates is shown at figure F4. Coordinate errors are visible as peak shifts.

**Table:**

> G $\lt -$ sfobs(hidx, sdata, ntrialP=10)
>
># Calculated amplitudes
> Fm[2 : 6]
[1] 6.289007 12.500798 9.582935 3.117837 2.375137
>
># Simulated amplitudes with errors
> Fobs $\lt -$ G$F
> Fobs [2 : 6]
[1] 6.241704 12.768149 9.598217 3.036468 2.62306
>
># Errors
> sFobs $\lt -$ $GsF > sFobs[2 : 6]
[1] 1.3411398 0.7941095 0.8766606 1.6118975 1.1133046

**Table:**

> set. seed (9195)
> G $\lt -$ sfobs(hidx, sdata, ntrial P=0, vx0err=0.4)
> Fobs $\lt -$ G$F
> sFobs $\lt -$ G$sF
> Fm [2 : 6]
[1] 6.289007 12.500798 9.582935 3.117837 2.375137
> Fobs [2 : 6]
[1] 7.307763 12.185575 9.088731 3.672122 2.202653
> sFobs [2 : 6]
[1] 2.1446024 2.5457612 1.5704811 0.7313995 0.2387590

### F.4. The breaking of Friedel's law

In section 5 it was mentioned that when a structure contains atoms which scatter anomalously in a significant way, then structure factors do not obey Friedel's law anymore. This can be nicely demonstrated using the strufac function. In the set of 1D molecules available within CRONE there are no structures including atoms scattering anomalously in any appreciable way. We can artificially create such a structure by replacing the carbon of thiocyanate with an iron atom:

**Table:**

># Iron has Z=26
> sdata$Z [2] $\lt -$ 26
>
># Reduce thermal vibration
> sdata$B [2] $\lt -$ 3.0
> print (sdata)
$x0
[1] 1.000 2.813 3.969
$Z
[1] 16 26 7
$B
[1] 5.000 3.000 11.429
$occ
[1] 1 1 1

Next, we can find out at which wavelength the anomalous scattering is expected to produce the largest differences in structure factors’ amplitudes, using function fluorescent_scan:

**Table:**

> print (fluorescent_scan (‘Fe’) $lambda)
[1] 1.742797

For wavelength close to this value the effect of anomalous scattering will be relatively large. We will, now, calculate structure factors where the anomalous scattering is switched off, so that the corresponding Friedel's pairs are exactly equal:

**Table:**

># Positive and negative h to show Friedel's pairs
> hidx $\lt --$ 10:10
>
># Exact structure factors: no anomalous effect
> ftmp1 $\lt -$ strufac (hidx, sdata, lbda=1.0, anoflag=FALSE) > modsF1 $\lt -$ ftmp1$Fmod
>
># Friedel's pair h =−1, h=1
> print (paste(modsF1[10], modsF1[12]))
[1] ‘15.682277494909 15.682277494909’
>
># Friedel's pair h= −2, h=2
> print(paste(modsF1[9], modsF1[13]))
[1] ‘18.0288655269983 18.0288655269983’

When the anomalous scattering is not switched off, but structure factors are calculated at a wavelength far from 1.74 Å, Friedel's pairs will not be exactly equal, but will be approximately close. In fact, atoms always scatter anomalously, but the magnitude of such scattering is very small except at specific resonant wavelengths:

**Table:**

># Exact structure factors: no anomalous effect
> ftmp2 $\lt -$ strufac (hidx, sdata, lbda=1.0, anoflag=TRUE)
> modsF2 $\lt -$ ftmp2$Fmod
>
># Friedel's pair h= −1, h=1
> print (paste (modsF2[10], modsF2[12]))
[1] ‘15.8261334218521 15.6615216563831’
>
># Friedel's pair h= −2, h=2
> print (paste (modsF2[9], modsF2[13]))
[1] ‘17.8722603191626 18.3860646475774’

When structure factors’ calculation is carried out at 1.74 Å, the iron atom will resonate anomalously and increase the difference between Friedel's pairs:

**Table:**

># Exact structure factors: no anomalous effect
> ftmp3 $\lt -$ strufac(hidx, sdata, lbda=1.74, anoflag=TRUE)
> modsF3 $\lt -$ ftmp3$Fmod
>
># Friedel's pair h= −1, h=1
> print(paste(modsF3[10], modsF3[12]))
[1] ‘14.1965995692363 13.7285463084373’
>
># Friedel's pair h= −2, h=2
> print (paste (modsF3[9], modsF3[13]))
[1] ‘16.1298629197846 17.5396455968006’

For real structure factors derived from collected data, the differences in amplitude for Friedel's pairs can be concealed by experimental errors. In CRONE this effect can be explored with the help of function sfobs.
